# Two types of weight-dependent walks with a trap in weighted scale-free treelike networks

**DOI:** 10.1038/s41598-018-19959-x

**Published:** 2018-01-24

**Authors:** Meifeng Dai, Yue Zong, Jiaojiao He, Xiaoqian Wang, Yu Sun, Weiyi Su

**Affiliations:** 1 Institute of Applied System Analysis, Faculty of Science, Jiangsu University, Zhenjiang, 212013 P. R. China; 2 Department of Mathematics, Nanjing University, Nanjing, 210093 P. R. China

## Abstract

In this paper, we present the weighted scale-free treelike networks controlled by the weight factor *r* and the parameter *m*. Based on the network structure, we study two types of weight-dependent walks with a highest-degree trap. One is standard weight-dependent walk, while the other is mixed weight-dependent walk including both nearest-neighbor and next-nearest-neighbor jumps. Although some properties have been revealed in weighted networks, studies on mixed weight-dependent walks are still less and remain a challenge. For the weighted scale-free treelike network, we derive exact solutions of the average trapping time (ATT) measuring the efficiency of the trapping process. The obtained results show that ATT is related to weight factor *r*, parameter *m* and spectral dimension of the weighted network. We find that in different range of the weight factor *r*, the leading term of ATT grows differently, i.e., superlinearly, linearly and sublinearly with the network size. Furthermore, the obtained results show that changing the walking rule has no effect on the leading scaling of the trapping efficiency. All results in this paper can help us get deeper understanding about the effect of link weight, network structure and the walking rule on the properties and functions of complex networks.

## Introduction

The past decade has witnessed a great deal of activity devoted to complex networks by the scientific community, since a number of real-life systems in nature and society can be described by complex networks.

In recent years, an important discovery of the research on complex networks is that there are scale-free networks with degree distribution satisfying power-law distribution $P(k) \sim {k}^{-\gamma }$, where *P*(*k*) is the probability of *k* for any node. In the scale-free networks, very few nodes have a large number of connections (usually called hubs), and most nodes have small degrees, thus, the degree distribution of scale-free network is heterogeneous. Many real networks have attracted much attention, because these networks display a scale-free feature, such as the protein interaction network of yeast, computer network and so on. More specifically, (1) protein interaction network of yeast^1^: most proteins interact with few partners, but a small significant part of proteins, namely hubs, interact with many partners. (2) computer network^2,3^: a computer network consists of interconnected computers and devices. In computer networks, networked computing devices exchange data through a data link. The connections between nodes are established through wired media or wireless media. Information can be transferred from one device to another. For example, an office filled with computers can share files together on each individual device. Computer networks can range from a local network area to a wide regional network. To study these real networks, we next develop a mathematical framework and construct the weighted scale-free treelike networks in this paper. However, the real life networks do not have the ideal structure. Real life networks are more random than ideal mathematical model. It should be mentioned that we only studied the trapping problem in a theoretical model of weighted scale-free treelike networks, whether the conclusion also holds for random networks, which needs further investigations in their future research.

Especially, in existing studies of complex networks, an important issue is to uncover the influence of topological characteristics on the dynamical processes occurring on networks^4^. Among various dynamical processes, diffusion is one of the fundamental dynamical process, since they can simulate or express many other physical processes^5–7^. In general, there are three kinds of walks: random walk, weight-dependent walk and strength-dependent walk. Recently, in order to discover what topological characteristics of network have important effect on diffusion, a lot of work focus on the average trapping time (ATT) which is used to characterize the efficiency of diffusion in different networks^8–10^.

So far, studies mainly focus on finding out the impact of some structural properties on random walks or weight-dependent walks^11–13^. In the walking process, in addition on the network structure, the walking rule itself also has an impact on the efficiency of walking. So, in many applications, optimizing the step length and preference of the walker can make the walking efficiency more efficient^6,14,15^. In addition, it is well known that many real networks are intrinsically weighted, and the weighted network can give more detailed description of interaction between agents of corresponding systems. Some studies have shown that the weights of network can affect the network dynamics^10,16,17^.

Moreover, most existent works focus on nearest-neighbor weight-dependent walks, neglecting the role of non-nearest-neighbor hopping (mixed weight-dependent walks), which has been involved in some physical processes, such as exciton migration in crystals^18^ and the surface diffusion of adatoms^19^. And non-nearest-neighbor hopping has been used to investigate and improve the traffic system via complex network^20^. Nevertheless, in contrast to nearest-neighbor weight-dependent walks, there are few theoretical studies on the influence of mixed weight-dependent walks on the network, especially on the trapping problem. The analytical results of ATT for weighted networks on mixed weight-dependent walks have been far less reported. In particular, exhaustive analytical research about diffusion including walking rule on weighted networks is still missing. Thus, it is quite important and significant to further explore how weight distribution and walking rule affect diffusion on weighted networks.

The organization of this paper is as follows. In Section 2, we introduce the weighted scale-free treelike networks and weight-dependent walks with a trap, then we calculate the ATT for two types of weight-dependent walks, standard weight-dependent walks and mixed weight-dependent walks, respectively. In Section 3, we draw the conclusion about the the effect of link weight, network structure and the walking rule on the weighted scale-free treelike networks. In the last section we give methods.

## Results

### Construction and properties of weighted scale-free treelike networks

This section aims at constructing the weighted scale-free treelike networks, which are the deterministic networks.

In the real world, stochastic model is more appropriate characteristic of most networks, but it is difficult to comprehend the network mechanisms of interaction and connection among different nodes. Deterministic networks are constructed in a deterministic manner that reflecting the real system characteristics.

Intuited by scale-free binary networks^21^ and weighted tree networks^22^, the weighted scale-free treelike networks, denoted by *F*_*n*_ (*n* ≥ 0), are built iteratively.

Let *r* (0 < *r* ≤ 1) be a positive real number, and *m* (*m* ≥ 1) be a positive integer.

**Figure 1:**
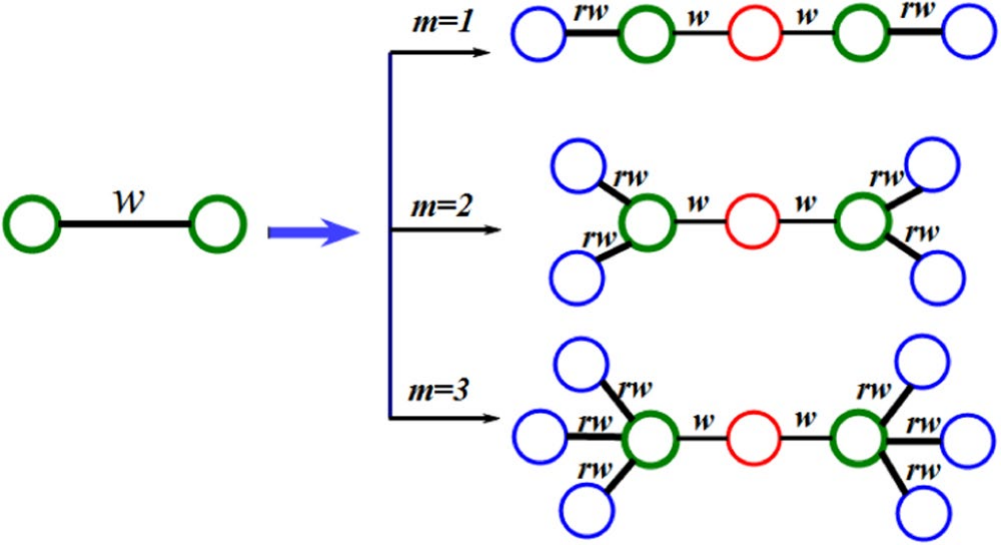
Iterative construction method on every edge for the weighted scale-free treelike networks for three special cases of *m* = 1, *m* = 2, and *m* = 3, where each blue node represents a new external node, while each red node stands for a new internal node.

1. At *n* = 0, let *F*_0_ be a base graph with two nodes connected by an edge with unitary weight.
2. For *n* ≥ 1, *F*_*n*_ is obtained from *F*_*n*−1_ by performing the following operations on every edge in *F*_*n*−1_.
  For each existing edge having two endpoints and edge weight *w* in *F*_*n*−1_, one can substitute a connected cluster on the right-hand side of arrow in Fig. 1 for each edge in *F*_*n*−1_ on the left-hand side of arrow in Fig. 1, as described below.
  (i) Use a path of 2 links long to replace each edge of *F*_*n*−1_, the two endpoints of the path is the same endpoints of the original edge, and the new node having an initial degree 2 is placed in the middle of path (This new node is called an internal node generated at *n*).
  (ii) Then, attach *m* new nodes with an initial degree 1 to each endpoint of the path, respectively. (The new 2*m* nodes are called external nodes generated at *n*).
  (iii) Every edge weight in *F*_*n*_ are scaled as shown in Fig. 1: The weight of two new edges connected the endpoint and the internal node in *F*_*n*_ are equal to the weight of the original edge in *F*_*n*−1_. The new 2*m* edges connected the endpoint and the external node are scaled by a weight factor *r*. Here *r* is called the weight factor.

**Figure 2:**
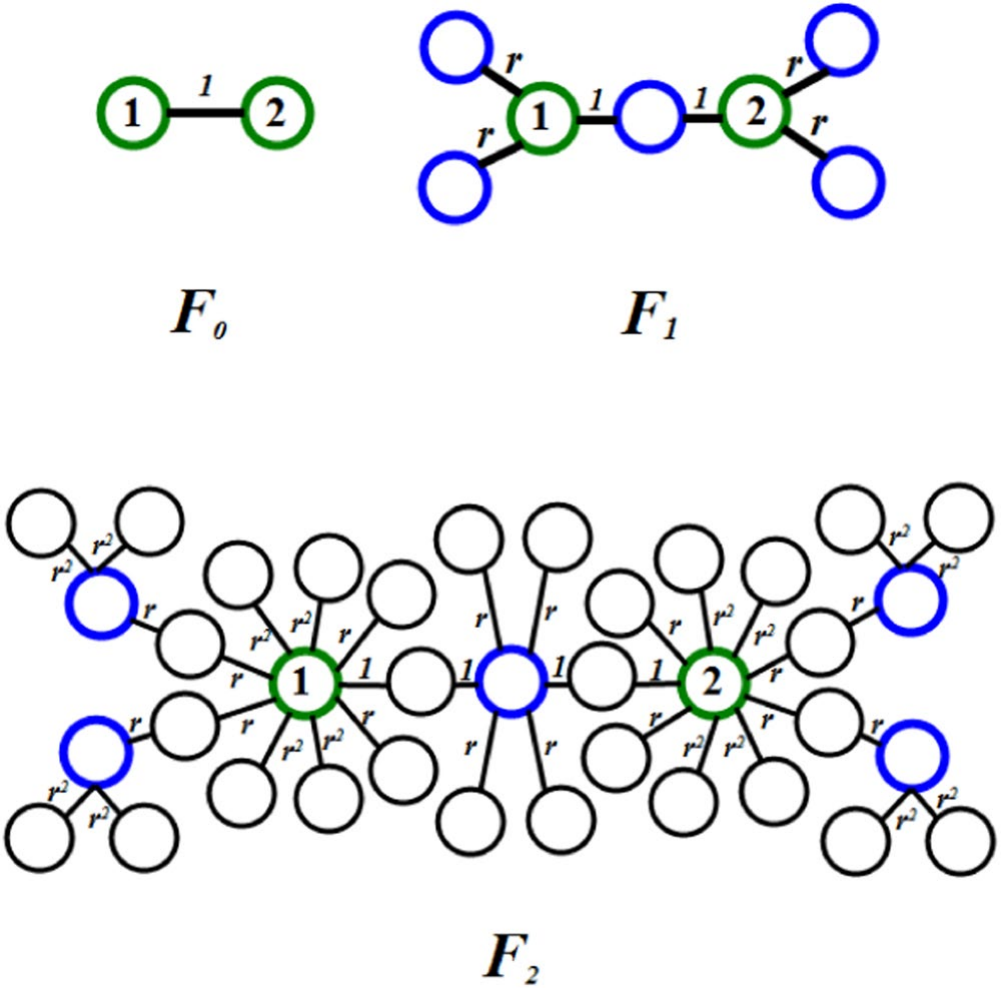
Iterative construction method for the weighted scale-free treelike networks *F*_*n*_(*m* = 2) from *n* = 0 to *n* = 2, where blue nodes are generated at *n* = 1, black nodes are generated at *n* = 2.

In Fig. 2, we schematically illustrate the process of the first three iterations for networks *F*_*n*_ when *m* = 2.

According to the structures of the weighted scale-free treelike networks, one can see that *F*_*n*_, is characterized by three parameters *n*, *m* and *r*. At each generation *n*_*i*_ (*n*_*i*_ ≥ 1), the number of newly introduced nodes is ${\bar{N}}_{{n}_{i}}=(2m+1){(2m+2)}^{{n}_{i}-1}$, where ${(2m+2)}^{{n}_{i}-1}$ nodes are internal nodes and the remaining $2m{(2m+2)}^{{n}_{i}-1}$ nodes are external nodes at generation *n*_*i*_. The total number of nodes in *F*_*n*_ is ${N}_{n}={\sum }_{{n}_{i}\mathrm{=0}}^{n}{\bar{N}}_{{n}_{i}}={(2m+2)}^{n}+1$. Let *k*_*i*_(*n*) be the degree of node *i*, which was generated at generation *n*_*i*_ in *F*_*n*_, (*n*_*i*_ ≥ 0), then ${k}_{i}(n)=2{(m+1)}^{n-{n}_{i}}$ if *i* is an internal node, and ${k}_{i}(n)={(m+1)}^{n-{n}_{i}}$ if *i* is an external node. Thus, the degree of node *i* increases from *k*_*i*_(*n*) to (*m* + 1)*k*_*i*_(*n*). Let *s*_*i*_(*n*) represent the strength of node *i*, then the strength of node *i* increases from *s*_*i*_(*n*) to (*mr* + 1)*s*_*i*_(*n*). The networks under consideration display the significant topological features as observed in various real systems. They are scale free with degree distribution $P(k) \sim {k}^{-\gamma }$, where *γ* = 1 + *ln*(2*m* + 2)/*ln*(*m* + 1)^23^. We have that the fractal dimension of the weighted scale-free treelike networks is ${d}_{f}=\frac{\mathrm{log}(2m+2)}{-\mathrm{log}\,r}$, 0 < *r* < 1. Moreover, the random-walk dimension of the weighted scale-free treelike networks is ${d}_{w}=\frac{\mathrm{log}(4mr+4)}{-\mathrm{log}\,r}$ (see Eq. (27)), and their spectral dimension

1 $${d}_{s}=2{d}_{f}/{d}_{w}=\frac{2\,\mathrm{log}\,\mathrm{(2}m+\mathrm{2)}}{\mathrm{log}\,\mathrm{(4}mr+\mathrm{4)}}\mathrm{.}$$

### The concept of weight-dependent walks with a trap

The peculiar architecture of the weighted scale-free treelike networks makes it worthwhile to study dynamical processes performing on them. In this section we consider two types of weight-dependent walks on weighted scale-free treelike networks *F*_*n*_ with a single trap fixed at one of its two initial nodes (for example, Node 1, see Fig. 2).

For convenience of description, let us denote by 1, 2 the two initial nodes in *F*_*n*_, and by 3, 4, …, *N*_*n*_ − 2 all other nodes except for the two initial nodes. Let *T*_*ij*_(*n*) represent the mean first-passage time (MFPT) from node *i* to *j* in *F*_*n*_, which is the expected time taken by a walker starting from *i* to first arrive at *j*. The highly desirable quantity related to the trapping problem is the average trapping time (ATT). Trapping time (TT) is defined as the expected time spent by a walker starting from a source node to first reach the trap node. Thus, the ATT is the average of trapping time over all non-trap starting nodes. Let *T*_*i*_(*n*) represent the TT for a walker starting from node *i* to first reach the trap node in *F*_*n*_. Then, the ATT, denoted by 〈*T*〉_*n*_, is the mean of *T*_*i*_(*n*) over all starting nodes other than the trap in *F*_*n*_^24^, which is given by

2 $${\langle T\rangle }_{n}=\frac{1}{{N}_{n}-1}\sum _{i\mathrm{=2}}^{{N}_{n}}{T}_{i}(n\mathrm{).}$$

Below we will introduce two types of weight-dependent walks, standard weight-dependent walk and mixed weight-dependent walk, performed on weighted scale-free treelike networks *F*_*n*_ and study the ATT for two types of weight-dependent walks, respectively.

### Leading scalings of ATT for standard weight-dependent walks on weighted scale-free treelike networks

The standard weight-dependent walk is a classical weight-dependent walk. In the process of standard weight-dependent walks, at each time step, the walker jumps from its current location *i* to one of its direct neighbors *j* with the transition probability ${P}_{i\to j}(n)=\frac{{w}_{ij}}{{s}_{i}(n)},$ where *w*_*ij*_ is the weight of edge linking node *i* and node *j*, *s*_*i*_(*n*) is the strength of node *i*. According to the construction algorithm of *F*_*n*_, we first derive a useful relation governing the evolution for *T*_*i*_(*n*) at generation *n*.

For an arbitrary node *i* in *F*_*n*−1_, after one generation, the degree of node *i* increases from *k*_*i*_(*n* − 1) to (*m* + 1)*k*_*i*_(*n* − 1), the strength of node *i* increases from *s*_*i*_(*n* − 1) to (*mr* + 1)*s*_*i*_(*n* − 1). In addition, (*m* + 1)*k*_*i*_(*n* − 1) neighbors of node *i* are new nodes created at generation *n*. Thereinto, *mk*_*i*_(*n* − 1) new nodes are external nodes, the rest *k*_*i*_(*n* − 1) neighbors are internal nodes at generation *n*. We can prove that (see Methods)

3 $${T}_{i}(n)=\mathrm{(4}mr+\mathrm{4)}{T}_{i}(n-\mathrm{1),}$$

which is a basic feature of the standard weight-dependent walks on *F*_*n*_ and helpful for the following derivation of the exact solutions of the average trapping time (ATT).

In order to obtain the 〈*T*〉_*n*_, from Eq. (2), we need to calculate the sum of *T*_*i*_(*n*) for all nodes in *F*_*n*_, denoted by ${T}_{n,total}^{n}$, and Eq. (2) can be written as

4 $${\langle T\rangle }_{n}=\frac{1}{{N}_{n}-1}{T}_{n,total}^{n}\mathrm{.}$$

Thus, in order to obtain the average trapping time in *F*_*n*_, the problem of determining 〈*T*〉_*n*_ is evolved into calculating ${T}_{n,total}^{n}$.

For convenience of description, the node set denoted by Ω_*n*_ of *F*_*n*_ is written as the union of three disjoint subsets ${{\rm{\Omega }}}_{n}={{\rm{\Omega }}}_{n-1}\cup {\bar{{\rm{\Omega }}}}_{{n}_{ext}}\cup {\bar{{\rm{\Omega }}}}_{{n}_{int}}$. Ω_*n*−1_ is the set of nodes existed at generation *n* − 1, ${\bar{{\rm{\Omega }}}}_{{n}_{ext}}$ is the set of external new nodes generated at *n*, the rest ${\bar{{\rm{\Omega }}}}_{{n}_{int}}$ is the set of internal new nodes generated at *n*. Let ${\bar{{\rm{\Omega }}}}_{n}={\bar{{\rm{\Omega }}}}_{{n}_{ext}}\cup {\bar{{\rm{\Omega }}}}_{{n}_{int}}$, then ${{\rm{\Omega }}}_{n}={{\rm{\Omega }}}_{n-1}\cup {\bar{{\rm{\Omega }}}}_{n}$. Notice that $|{\bar{{\rm{\Omega }}}}_{{n}_{ext}}|=2m{\mathrm{(2}m+\mathrm{2)}}^{n-1}$ and $|{\bar{{\rm{\Omega }}}}_{{n}_{int}}|={\mathrm{(2}m+\mathrm{2)}}^{n-1}$.

Next, we define four intermediate variables for 1 ≤ *m* ≤ *n*:

5 $$\begin{array}{cc}{T}_{m,total}^{n}=\sum _{i\in {{\rm{\Omega }}}_{m}}{T}_{i}(n), & {\bar{T}}_{m,total}^{n}=\sum _{i\in {\bar{{\rm{\Omega }}}}_{m}}{T}_{i}(n),\\ {\bar{T}}_{m,ext}^{n}=\sum _{i\in {\bar{{\rm{\Omega }}}}_{{m}_{ext}}}{T}_{i}(n), & {\bar{T}}_{m,int}^{n}=\sum _{i\in {\bar{{\rm{\Omega }}}}_{{m}_{int}}}{T}_{i}(n\mathrm{).}\end{array}$$

We have

6 $$\begin{array}{rcl}{T}_{n,total}^{n} & = & {T}_{n-\mathrm{1,}total}^{n}+{\bar{T}}_{n,total}^{n}\\  & = & {T}_{n-\mathrm{1,}total}^{n}+{\bar{T}}_{n,int}^{n}+{\bar{T}}_{n,ext}^{n}\mathrm{.}\end{array}$$

On the one hand, using the relation *T*_*i*_(*n*) = (4*mr* + 4)*T*_*i*_(*n* − 1), Eq. (6) can be written as

7 $${T}_{n,total}^{n}=\mathrm{(4}mr+\mathrm{4)}{T}_{n-\mathrm{1,}total}^{n-1}+{\bar{T}}_{n,int}^{n}+{\bar{T}}_{n,ext}^{n}\mathrm{.}$$

On the other hand, to find ${T}_{n,total}^{n}$, we need to calculate the two quantities ${\bar{T}}_{n,ext}^{n}$ and ${\bar{T}}_{n,int}^{n}$.

By construction, we can prove that (see Methods)

8 $${\bar{T}}_{n,ext}^{n}=2m{\bar{T}}_{n,int}^{n}\mathrm{.}$$

Then, from Eqs (7) and (8), we can get

9 $${T}_{n,total}^{n}=\mathrm{(4}mr+\mathrm{4)}{T}_{n-\mathrm{1,}total}^{n-1}+\frac{2m+1}{2m}{\bar{T}}_{n,ext}^{n}\mathrm{.}$$

Thus, the problem of determining ${T}_{n,total}^{n}$ is evolved into calculating ${\bar{T}}_{n,ext}^{n}$.

After some discussion and simplification, then we can get the recursion formula of ${\overline{T}}_{n,ext}^{n}$ here we just give a result and put off the proof and details in Methods. In Methods, we derive that ${\overline{T}}_{n,ext}^{n}$ obeys the following recursive relation:

10 $${\bar{T}}_{n+\mathrm{1,}ext}^{n+1}=\mathrm{8(}mr+\mathrm{1)(}m+\mathrm{1)}{\bar{T}}_{n,ext}^{n}-2m\mathrm{(2}mr+\mathrm{1)(2}m+{\mathrm{2)}}^{n}\mathrm{.}$$

Considering the initial condition ${\bar{T}}_{\mathrm{1,}ext}^{1}=4{m}^{2}r+6m$, Eq. (10) is solved by induction to obtain

11 $${\bar{T}}_{n,ext}^{n}=\frac{m\mathrm{(2}mr+\mathrm{1)}}{(m+\mathrm{1)(4}mr+\mathrm{3)}}{\mathrm{(2}m+\mathrm{2)}}^{n}+\frac{2m(mr+\mathrm{1)}}{(m+\mathrm{1)(4}mr+\mathrm{3)}}\times {8}^{n}{(mr+\mathrm{1)}}^{n}{(m+\mathrm{1)}}^{n}\mathrm{.}$$

Thus, substituting Eq. (11) into Eq. (9) yields, we have

12 $$\begin{array}{c}{T}_{n,total}^{n}=\mathrm{(4}mr+\mathrm{4)}{T}_{n-\mathrm{1,}total}^{n-1}+\frac{\mathrm{(2}m+\mathrm{1)(2}mr+\mathrm{1)}}{\mathrm{2(}m+\mathrm{1)(4}mr+\mathrm{3)}}{\mathrm{(2}m+\mathrm{2)}}^{n}\\ \,\,\,\,\,\,\,\,\,\,\,\,\,\,+\frac{\mathrm{(2}m+\mathrm{1)(}mr+\mathrm{1)}}{(m+\mathrm{1)(4}mr+\mathrm{3)}}\times {8}^{n}{(mr+\mathrm{1)}}^{n}{(m+\mathrm{1)}}^{n}\mathrm{.}\end{array}$$

Thus, from Eqs (1), (4) and (12), in Methods, we can prove that for very large network,

13 $${\langle T\rangle }_{n} \sim \{\begin{array}{ll}{({N}_{n})}^{1+\frac{ln\frac{2mr+2}{m+1}}{ln\mathrm{(2}m+\mathrm{2)}}}, & {\rm{if}}\,r\ne \frac{m-1}{2m}\,{\rm{and}}\,0 < r\le \mathrm{1,}\\ {N}_{n}, & {\rm{if}}\,r=\frac{m-1}{2m} > \mathrm{0,}\end{array}$$

where $1+\frac{ln\frac{2mr+2}{m+1}}{ln(2m+2)}$ is equal to the $\frac{2}{{d}_{s}}$ (*d*_*s*_ is the spectral dimension).

We find that if *r* = 1,

14 $${\langle T\rangle }_{n} \sim {({N}_{n})}^{1+\frac{ln2}{ln\mathrm{(2}m+\mathrm{2)}}},$$

which coincides with the 〈*T*〉_*n*_ in ref.^25^.

### Leading scalings of ATT for mixed weight-dependent walks on weighted scale-free treelike networks

The mixed weight-dependent walk is a specific and selective weight-dependent walk. In the process of mixed weight-dependent walks, in a time step, the walker is not only allowed to walk to its nearest neighbor nodes, but also can walk directly to its next-nearest neighbor with a certain probability. We define the rule of mixed weight-dependent walks in *F*_*n*_ as follows:

1. If the current location of the walker is an old node created before generation *n*, the walker can jump to nearest neighbor nodes with probabilities *θ*, and next-nearest neighbor nodes with probabilities 1 − *θ* (0 ≤ *θ* ≤ 1). The walker jumps to one of nearest neighbor nodes (next-nearest neighbor nodes) with the standard weight-dependent walks.
2. If the current location of the walker is a new node created at generation *n*, then the walker can only jump to one of the nearest neighbor nodes with the standard weight-dependent walks^21^.

For mixed weight-dependent walks in *F*_*n*_, the walker jumps from node *i* to node *j* with the transition probability *P*_*i* → *j*_(*n*) as follows.

15 $${P}_{i\to j}(n)=\{\begin{array}{ll}\frac{\theta {w}_{ij}(n)}{{s}_{i}(n)}, & {\rm{if}}\,i\in {{\rm{\Omega }}}_{n-1},j\in {\bar{{\rm{\Omega }}}}_{n},\\ \frac{\mathrm{(1}-\theta ){w}_{ij}(n-\mathrm{1)}}{{s}_{i}(n-\mathrm{1)}}, & {\rm{if}}\,i\in {{\rm{\Omega }}}_{n-1},j\in {{\rm{\Omega }}}_{n-1},\\ \frac{{w}_{ij}(n)}{{s}_{i}(n)}, & {\rm{if}}\,i\in {\bar{{\rm{\Omega }}}}_{n},j\in {{\rm{\Omega }}}_{n}\mathrm{.}\end{array}$$

when *θ* = 1, the mixed weight-dependent walk becomes the standard weight-dependent walk in *F*_*n*_.

Next, we will study the mixed weight-dependent walks in *F*_*n*_ with a trap located at one of its two initial nodes (for example, Node 1, see Fig. 2), and calculate the average trapping time (ATT) for the mixed weight-dependent walks. For the convenience of computation, we continue to use the variables defined for the standard weight-dependent walks to express the corresponding meaning in this section, in other words, we use the symbols *M*_*i*_(*n*), ${M}_{n,total}^{n}$, ${\overline{M}}_{n,total}^{n}$, and 〈*M*〉_*n*_ to represent *T*_*i*_(*n*), ${T}_{n,total}^{n}$, ${\overline{T}}_{n,total}^{n}$, and 〈*T*〉_*n*_ obtained for the standard weight-dependent walks in *F*_*n*_, respectively. Below we will have a useful scaling relation between and *T*_*i*_(*n* + 1) and *M*_*i*_(*n*) (Proof in Method).

16 $${T}_{i}(n+\mathrm{1)=}\frac{\mathrm{2(}mr+\mathrm{1)(}\theta +\mathrm{1)}}{2mr+2-\theta -2mr\theta }{M}_{i}(n),$$

which is a useful relation for the following derivation of the exact solution to ATT 〈*T*〉_*n*_ for the mixed weight-dependent walks. For *θ* = 1, Eq. (16) becomes *M*_*i*_(*n* + 1) = (4*mr* + 4)*M*_*i*_(*n*), which is consistent with the result obtained for the standard weight-dependent walks.

From Eq. (4), we can know, in order to determine 〈*T*〉_*n*_, we can obtain the ${T}_{n,total}^{n}$ that obeys the following relation.

17 $$\begin{array}{rcl}{T}_{n,total}^{n} & = & {T}_{n-\mathrm{1,}total}^{n}+{\bar{T}}_{n,total}^{n}\\  & = & \frac{\mathrm{2(}mr+\mathrm{1)(}\theta +\mathrm{1)}}{2mr+2-\theta -2mr\theta }{M}_{n-\mathrm{1,}total}^{n-1}+{\overline{T}}_{n,total}^{n}\mathrm{.}\end{array}$$

Then, we focus on the quantity ${\bar{T}}_{n,total}^{n}$ to obtain the ${T}_{n,total}^{n}$.

By construction, we can prove that (see Methods)

18 $$\begin{array}{rcl}{\bar{T}}_{n,total}^{n} & = & |{\bar{{\rm{\Omega }}}}_{n}|+\sum _{i\in {{\rm{\Omega }}}_{n-1}}[(m+\frac{1}{2}){k}_{i}(n-\mathrm{1)}\times {T}_{i}(n)]\\  & = & |{\bar{{\rm{\Omega }}}}_{n}|+(m+\frac{1}{2})\sum _{i\in {{\rm{\Omega }}}_{n-1}}[{k}_{i}(n-\mathrm{1)}\frac{\mathrm{2(}mr+\mathrm{1)(}\theta +\mathrm{1)}}{2mr+2-\theta -2mr\theta }{M}_{i}(n-\mathrm{1)}]\mathrm{.}\end{array}$$

When *θ* = 1, Eq. (18) can be written as

19 $${\overline{M}}_{n,total}^{n}=|{\overline{{\rm{\Omega }}}}_{n}|+(m+\frac{1}{2})\sum _{i\in {{\rm{\Omega }}}_{n-1}}({k}_{i}(n-\mathrm{1)(4}mr+\mathrm{4)}{M}_{i}(n-\mathrm{1)).}$$

Combining Eqs (18) and (19), we can get

20 $$\frac{{\bar{T}}_{n,total}^{n}-|{\bar{{\rm{\Omega }}}}_{n}|}{\frac{\mathrm{2(}mr+\mathrm{1)(}\theta +\mathrm{1)}}{2mr+2-\theta -2mr\theta }}=\frac{{\bar{M}}_{n,total}^{n}-|{\bar{{\rm{\Omega }}}}_{n}|}{\mathrm{(4}mr+\mathrm{4)}}\mathrm{.}$$

According to $|{\bar{{\rm{\Omega }}}}_{n}|=\mathrm{(2}m+\mathrm{1)(2}m+{\mathrm{2)}}^{n-1}$, Eq. (20) can be simplified as

21 $${\bar{T}}_{n,total}^{n}=\frac{\theta +1}{4mr+4-2\theta -4mr\theta }{\bar{M}}_{n,total}^{n}+\frac{{\mathrm{(2}m+\mathrm{2)}}^{n-1}\mathrm{(2}m+\mathrm{1)(4}mr+\mathrm{3)(1}-\theta )}{4mr+4-2\theta -4mr\theta }\mathrm{.}$$

In addition, we have

22 $${\bar{M}}_{n,total}^{n}={M}_{n,total}^{n}-{M}_{n-\mathrm{1,}total}^{n}={M}_{n,total}^{n}-\mathrm{(4}mr+\mathrm{4)}{M}_{n-\mathrm{1,}total}^{n-1}\mathrm{.}$$

Plugging Eqs (21) and (22) into Eq. (17), we can have

23 $${T}_{n,total}^{n}=\frac{\theta +1}{4mr+4-2\theta -4mr\theta }{M}_{n,total}^{n}+\frac{{\mathrm{(2}m+\mathrm{2)}}^{n-1}\mathrm{(2}m+\mathrm{1)(4}mr+\mathrm{3)(1}-\theta )}{4mr+4-2\theta -4mr\theta }\mathrm{.}$$

Using Eq. (4) and *N*_*n*_ = (2*m* + 2)^*n*^ + 1, we can get the accurate formula for the ATT 〈*T*〉_*n*_.

24 $$\begin{array}{rcl}{\langle T\rangle }_{n} & = & \frac{1}{{N}_{n}-1}{T}_{n,total}^{n}=\frac{1}{{\mathrm{(2}m+\mathrm{2)}}^{n}}{T}_{n,total}^{n}\\  & = & \frac{\theta +1}{4mr+4-2\theta -4mr\theta }{\langle M\rangle }_{n}+\frac{\mathrm{(2}m+\mathrm{1)(4}mr+\mathrm{3)(1}-\theta )}{\mathrm{(2}m+\mathrm{2)(4}mr+4-2\theta -4mr\theta )}\mathrm{.}\end{array}$$

Then, we can get the ATT for the mixed weight-dependent walks for very large netwgrk (details in Method),

25 $${\langle T\rangle }_{n} \sim \{\begin{array}{ll}{({N}_{n})}^{1+\frac{ln\frac{2mr+2}{m+1}}{ln\mathrm{(2}m+\mathrm{2)}}}\mathrm{,\ } & {\rm{if}}\,r\ne \frac{m-1}{2m}\,{\rm{and}}\,0 < r\le 1,\\ {N}_{n}, & {\rm{if}}\,r=\frac{m-1}{2m} > 0.\end{array}$$

## Discussion

Therefore, the leading scaling of the ATT for standard weight-dependent walks and mixed weight-dependent walks on network *F*_*n*_ have the same law as follow:

When $r=\frac{m-1}{2m} > 0$, the average trapping time ATT grows linear with the network size *N*_*n*_. When $r\ne \frac{m-1}{2m}$ and 0 < *r* ≤ 1, ATT grows as a power-law function of network size *N*_*n*_, with the exponent $1+\frac{ln\frac{2mr+2}{m+1}}{ln(2m+2)}$, which depends on parameters *m* and *r*. If the parameter *m* is constant, and *r* grows from 0 to 1, the exponent is increasing. Thereinto, when $0 < r < \frac{m-1}{2m}$, the exponent $1+\frac{ln\frac{2mr+2}{m+1}}{ln(2m+2)}$ is smaller than 1. In the limit of the large network, the ATT grows sublinearly with the network size. When $\frac{m-1}{2m} < r\le 1$, the exponent $1+\frac{ln\frac{2mr+2}{m+1}}{ln(2m+2)}$ is greater than 1. In the limit of the large network, the ATT grows superlinearly with the network size. Thus, the smaller value of *r*, the more efficient the trapping process is. Therefore, the transportation efficiency could be controlled by adjusting the weight factor *r* in weighted scale-free treelike networks *F*_*n*_, and the weight factor *r* has an important effect on the average trapping time of weighted networks *F*_*n*_.

Moreover, the exponent $1+\frac{ln\frac{2mr+2}{m+1}}{ln(2m+2)}$ is equal to the $\frac{2}{{d}_{s}}$, which is related to the spectral dimension of the weighted scale-free treelike networks for the standard weight-dependent walks. However, the exponent is independent of parameter *θ*, so, the parameter *θ* has a negligible effect on the leading scaling of ATT. The heterogeneity of weight distribution and the change of weight factor *r* can affect the communication efficiency of the network. So, our study is meaningful, our model has advantage over the binary model and provides a useful tool in characterizing real complex systems.

## Methods

### Proof of the recursive relation between *T*_*i*_(*n* − 1) and *T*_*i*_(*n*) for standard weight-dependent walks

For an arbitrary node *i* in *F*_*n*−1_, let *X* be the MFPT starting from the node *i* to any of its *k*_*i*_(*n* − 1) old neighbors, which are directly connected to node *i* at generation *n* − 1. Let *Y* be the MFPT from any of *k*_*i*_(*n* − 1) internal new neighbors of node *i* to one of its *k*_*i*_(*n* − 1) old neighbors. Let *Z* be the MFPT from any of *mk*_*i*_(*n* − 1) external new neighbors of node *i* to one of its *k*_*i*_(*n* − 1) old neighbors. We can establish the following relations among *X*, *Y*, *Z*.

26 $$\{\begin{array}{rcl}X & = & \frac{mr}{mr+1}\mathrm{(1}+Z)+\frac{1}{mr+1}\mathrm{(1}+Y),\\ Y & = & \frac{1}{2}+\frac{1}{2}\mathrm{(1}+X),\\ Z & = & 1+X\mathrm{.}\end{array}$$

Solving Eq. (26), we can get *X* = 4*mr* + 4. Therefore, when the network grows from generation *n* − 1 to *n*, the MFPT between any pair of nodes *i* and *j* in *F*_*n*−1_ increases by a factor of *X* = 4*mr* + 4. Thus, we have

27 $${T}_{i}(n)=\mathrm{(4}mr+\mathrm{4)}{T}_{i}(n-\mathrm{1).}$$

### Proof of relation between ${\bar{{\boldsymbol{T}}}}_{{\boldsymbol{n}}{\boldsymbol{,}}{\boldsymbol{e}}{\boldsymbol{x}}{\boldsymbol{t}}}^{{\boldsymbol{n}}}$ and ${\bar{{\boldsymbol{T}}}}_{n,{\boldsymbol{i}}{\boldsymbol{n}}{\boldsymbol{t}}}^{{\boldsymbol{n}}}$ for standard weight-dependent walks

**Figure 3:**
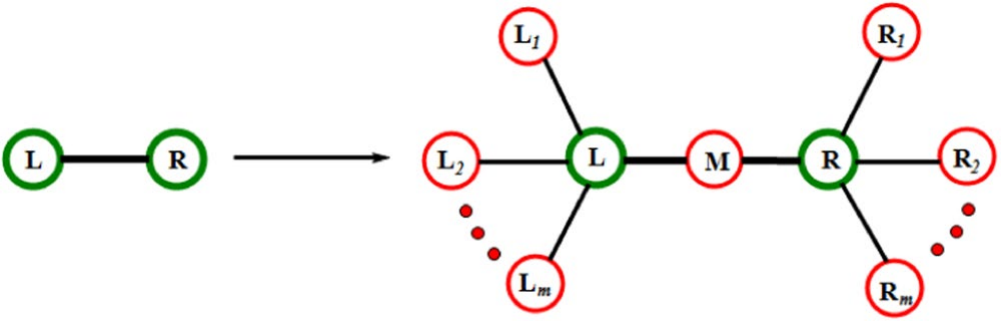
Illustration showing the relation of the trapping times for new nodes and old nodes connected by an edge generating the new nodes.

By construction of weighted scale-free treelike networks, we know that at given generation *n* − 1, each edge connecting two nodes *L* and *R* will generate 2*m* + 1 new nodes in the next generation (see Fig. 3): one internal node *M* generated at *n* connects node *L* and node *R*, *m* external nodes *L*_1_, …, *L*_*m*_ generated at *n* connect to node *L*, *m* external nodes *R*_1_, …, *R*_*m*_ generated at *n* connect to node *R*. We can establish the following relations among *T*_*L*_(*n*), *T*_*R*_(*n*), *T*_*M*_(*n*), ${T}_{{L}_{i}}(n)$, ${T}_{{R}_{i}}(n)$ (*i* = 1, …, *m*).

28 $$\{\begin{array}{ll}{T}_{{L}_{i}}(n) & =\,1+{T}_{L}(n\mathrm{),\ \ \ }\\ {T}_{{R}_{i}}(n) & =\,1+{T}_{R}(n\mathrm{),\ \ \ \ \ \ \ \ \ \ \ \ \ \ \ \ \ \ \ \ \ }\\ {T}_{M}(n) & =\,\frac{1}{2}\mathrm{(1}+{T}_{L}(n))+\frac{1}{2}\mathrm{(1}+{T}_{R}(n\mathrm{)).}\end{array}$$

Solving Eq. (28), we can get

29 $$\sum _{i\mathrm{=1}}^{m}[{T}_{{L}_{i}}(n)+{T}_{{R}_{i}}(n)]=2m{T}_{M}(n\mathrm{).}$$

Summing Eq. (29) over all the edges pre-existing in *F*_*n*_, we find that,

30 $${\bar{T}}_{n,ext}^{n}=2m{\bar{T}}_{n,int}^{n}\mathrm{.}$$

### Derivation for the recursive relation between ${\bar{{\boldsymbol{T}}}}_{{\boldsymbol{n,}}{\boldsymbol{e}}{\boldsymbol{x}}{\boldsymbol{t}}}^{{\boldsymbol{n}}}$ and ${\bar{{\boldsymbol{T}}}}_{{\boldsymbol{n}}+{\boldsymbol{1,}}{\boldsymbol{e}}{\boldsymbol{x}}{\boldsymbol{t}}}^{n+{\boldsymbol{1}}}$ for standard weight-dependent walks

Consider an arbitrary external node *i*_*ext*_ in *F*_*n*_, which was added at generation *n* and linked to an old node *i*, we have

31 $${T}_{{i}_{ext}}(n)=1+{T}_{i}(n\mathrm{).}$$

Since a walker starting from *i*_*ext*_ will for certain reach *i* after one jump. Note that Eq. (31) holds for any node pair consisting of an old node and one of its new external adjacent nodes.

Notice that

32 $${{\rm{\Omega }}}_{n}=({\bar{{\rm{\Omega }}}}_{{n}_{ext}}\cup {\bar{{\rm{\Omega }}}}_{{n}_{int}})\cup ({\bar{{\rm{\Omega }}}}_{n-{1}_{ext}}\cup {\bar{{\rm{\Omega }}}}_{n-{1}_{int}})\cup \cdots \cup ({\bar{{\rm{\Omega }}}}_{{1}_{ext}}\cup {\bar{{\rm{\Omega }}}}_{{1}_{int}})\cup {{\rm{\Omega }}}_{0},$$

and

33 $${k}_{i}(n)=\{\begin{array}{ll}{(m+\mathrm{1)}}^{n}, & {\rm{if}}\,i\in {{\rm{\Omega }}}_{0},\\ {(m+\mathrm{1)}}^{n-t}, & {\rm{if}}\,i\in {\bar{{\rm{\Omega }}}}_{{t}_{ext}},\\ \mathrm{2(}m+{\mathrm{1)}}^{n-t}, & {\rm{if}}\,i\in {\bar{{\rm{\Omega }}}}_{{t}_{int}}\mathrm{.}\end{array}$$

From Eqs (30), (31), (32) and (33), we obtain

34 $$\begin{array}{rcl}{\overline{T}}_{n+\mathrm{1,}ext}^{n+1} & = & |{\bar{{\rm{\Omega }}}}_{n+{1}_{ext}}|+\sum _{i\in {{\rm{\Omega }}}_{n}}[m{k}_{i}(n)\times {T}_{i}(n+\mathrm{1)}]\\  & = & |{\bar{{\rm{\Omega }}}}_{n+{1}_{ext}}|+m({\bar{T}}_{n,ext}^{n+1}+2{\bar{T}}_{n,int}^{n+1})+m(m+\mathrm{1)}({\bar{T}}_{n-\mathrm{1,}ext}^{n+1}+2{\bar{T}}_{n-\mathrm{1,}int}^{n+1})\\  &  & \begin{array}{c}+\cdots +m{(m+\mathrm{1)}}^{n-1}({\bar{T}}_{\mathrm{1,}ext}^{n+1}+2{\bar{T}}_{\mathrm{1,}int}^{n+1})\\ +\,m{(m+\mathrm{1)}}^{n}({T}_{1}(n+\mathrm{1)}+{T}_{2}(n+\mathrm{1))}\end{array}\\  & = & 2m{\mathrm{(2}m+\mathrm{2)}}^{n}+(m+\mathrm{1)}{\bar{T}}_{n,ext}^{n+1}+{(m+\mathrm{1)}}^{2}{\bar{T}}_{n-\mathrm{1,}ext}^{n+1}\\  &  & +\cdots +{(m+\mathrm{1)}}^{n}{\bar{T}}_{\mathrm{1,}ext}^{n+1}+m{(m+\mathrm{1)}}^{n}({T}_{1}(n+\mathrm{1)}+{T}_{2}(n+\mathrm{1)).}\end{array}$$

Similarity, we also can write the formula for ${\bar{T}}_{n,ext}^{n}$ as

35 $$\begin{array}{rcl}{\bar{T}}_{n,ext}^{n} & = & 2m{\mathrm{(2}m+\mathrm{2)}}^{n-1}+(m+\mathrm{1)}{\bar{T}}_{n-\mathrm{1,}ext}^{n}+{(m+\mathrm{1)}}^{2}{\bar{T}}_{n-\mathrm{2,}ext}^{n}\\  &  & +\cdots +{(m+\mathrm{1)}}^{n-1}{\bar{T}}_{\mathrm{1,}ext}^{n}+m{(m+\mathrm{1)}}^{n-1}({T}_{1}(n)+{T}_{2}(n\mathrm{)).}\end{array}$$

Using *T*_*i*_(*n* + 1) = (4*mr* + 4)*T*_*i*_(*n*), Eq. (34) can be written as

36 $$\begin{array}{rcl}{\bar{T}}_{n+\mathrm{1,}ext}^{n+1} & = & 2m{\mathrm{(2}m+\mathrm{2)}}^{n}+\mathrm{(4}mr+\mathrm{4)(}m+\mathrm{1)}{\bar{T}}_{n,ext}^{n}+\mathrm{(4}mr+\mathrm{4)(}m+{\mathrm{1)}}^{2}{\bar{T}}_{n-\mathrm{1,}ext}^{n}\\  &  & +\cdots +\mathrm{(4}mr+\mathrm{4)(}m+{\mathrm{1)}}^{n}{\bar{T}}_{\mathrm{1,}ext}^{n}+m\mathrm{(4}mr+\mathrm{4)(}m+{\mathrm{1)}}^{n}({T}_{1}(n)+{T}_{2}(n\mathrm{)).}\end{array}$$

From Eqs (35) and (34), we can get

37 $${\bar{T}}_{n+\mathrm{1,}ext}^{n+1}=\mathrm{8(}mr+\mathrm{1)(}m+\mathrm{1)}{\bar{T}}_{n,ext}^{n}-2m\mathrm{(2}mr+\mathrm{1)(2}m+{\mathrm{2)}}^{n}\mathrm{.}$$

### Proof of leading scalings of ATT for standard weight-dependent walks

From Eq. (12), use the initial value ${T}_{\mathrm{1,}total}^{1}=4{m}^{2}r+6mr+6m+7$, and notice that *r* (0 < *r* ≤ 1) is a positive real number. We will give a classified discussion about the range of the weight factor *r* to solve Eq. (12) in the following two cases:

*Case* 1: When *r* ≠ (*m* − 1)/(2*m*) and 0 < *r* ≤ 1,

38 $$\begin{array}{rcl}{T}_{n,total}^{n} & = & \frac{\mathrm{2(}mr+\mathrm{1)}}{4mr+3}\times {8}^{n}{(mr+\mathrm{1)}}^{n}{(m+\mathrm{1)}}^{n}\\  &  & -\,\frac{\mathrm{(2}mr+\mathrm{1)(2}m+\mathrm{1)}}{\mathrm{2(4}mr+\mathrm{3)(2}rm+1-m)}\times {\mathrm{(2}m+\mathrm{2)}}^{n}\\  &  & +\,(\frac{4{m}^{2}r+6mr+6m+7}{\mathrm{4(}mr+\mathrm{1)}}-\frac{\mathrm{4(}mr+\mathrm{1)(}m+\mathrm{1)}}{4mr+3}\\  &  & +\,\frac{\mathrm{(2}m+\mathrm{1)(2}mr+\mathrm{1)(}m+\mathrm{1)}}{\mathrm{4(}mr+\mathrm{1)(4}mr+\mathrm{3)(2}rm+1-m)})\times {4}^{n}{(mr+\mathrm{1)}}^{n}\mathrm{.}\end{array}$$

Inserting Eq. (38) into Eq. (4) and according to *N*_*n*_ = (2*m* + 2)^*n*^ + 1, we can obtain the exact dependence relation of ATT on the network size *N*_*n*_ and weight factor *r* for the standard weight-dependent walks as

39 $$\begin{array}{cc}{\langle T\rangle }_{n} & =\,\frac{1}{{N}_{n}-1}{T}_{n,total}^{n}=\frac{1}{{\mathrm{(2}m+\mathrm{2)}}^{n}}{T}_{n,total}^{n}\\  & \approx \,\frac{\mathrm{2(}mr+\mathrm{1)}}{4mr+3}\times {({N}_{n}-\mathrm{1)}}^{1+\frac{ln\frac{2mr+2}{m+1}}{ln\mathrm{(2}m+\mathrm{2)}}}\mathrm{.}\end{array}$$

*Case* 2: When *r* = (*m* − 1)/(2*m*) > 0,

40 $$\begin{array}{rcl}{T}_{n,total}^{n} & = & \frac{m+1}{2m+1}{\mathrm{(2}m+\mathrm{2)}}^{2n}+\frac{mn}{\mathrm{2(}m+\mathrm{1)}}{\mathrm{(2}m+\mathrm{2)}}^{n}\\  &  & +\,(\frac{4{m}^{2}r+6mr+5m+7}{2m+2}-\frac{\mathrm{2(}m+{\mathrm{1)}}^{2}}{2m+1}){\mathrm{(2}m+\mathrm{2)}}^{n}\mathrm{.}\end{array}$$

Inserting Eq. (40) into Eq. (4) and according to *N*_*n*_ = (2*m* + 2)^*n*^ + 1, we can obtain the exact dependence relation of ATT on the network size *N*_*n*_ for the standard weight-dependent walks as

41 $$\begin{array}{lc}{\langle T\rangle }_{n} & =\,\frac{1}{{N}_{n}-1}{T}_{n,total}^{n}\\  & \quad \approx \,\frac{m+1}{2m+1}({N}_{n}-\mathrm{1).}\end{array}$$

Thus, from above two cases, we can know that for very large network,

42 $${\langle T\rangle }_{n} \sim \{\begin{array}{ll}{({N}_{n})}^{1+\frac{ln\frac{2mr+2}{m+1}}{ln\mathrm{(2}m+\mathrm{2)}}}, & {\rm{if}}\,r\ne \frac{m-1}{2m}{\rm{and}}\,0 < r\le 1,\\ {N}_{n}, & {\rm{if}}\,r=\frac{m-1}{2m} > \mathrm{0.\ }\end{array}$$

### Proof of relation between *T*_*i*_(*n* + 1) and *M*_*i*_(*n*) for mixed weight-dependent walks

Similar to the standard weight-dependent walks in *F*_*n*_, according to the above rule of mixed weight-dependent walks and Eq. (15), we can have the following relations about *X*, *Y*, and *Z* for the mixed weight-dependent walks.

43 $$\{\begin{array}{rcl}X & = & \frac{\theta mr}{mr+1}\mathrm{(1}+Z)+\frac{\theta }{mr+1}\mathrm{(1}+Y)+\mathrm{(1}-\theta ),\\ Y & = & \frac{1}{2}+\frac{1}{2}\mathrm{(1}+X),\\ Z & = & 1+X.\end{array}$$

Solving Eq. (43), we can get $X=\frac{2(mr+1)(\theta +1)}{2mr+2-\theta -2mr\theta }$. Therefore, when the network grows from generation *n* to *n* + 1, the MFPT between any pair of nodes *i* and *j* in *F*_*n*_ increases by a factor of $\frac{2(mr+1)(\theta +1)}{2mr+2-\theta -2mr\theta }$. Thus, we have

44 $${T}_{i}(n+\mathrm{1)}=\frac{\mathrm{2(}mr+\mathrm{1)(}\theta +\mathrm{1)}}{2mr+2-\theta -2mr\theta }{M}_{i}(n\mathrm{).}$$

### Proof of analytic expression about ${\overline{T}}_{n,total}^{n}$ for mixed weight-dependent walks

Consider an arbitrary external node *i*_*ext*_ in *F*_*n*_, which generated at *n* and linked to an old node *i*, we have

45 $${T}_{{i}_{ext}}(n)=1+{T}_{i}(n\mathrm{).}$$

While for any internal node *l*_*int*_ in *F*_*n*_, which generated at *n* and connected to a pair old nodes *i* and *j*, we obtain

46 $${T}_{{l}_{int}}(n)=1+\frac{1}{2}{T}_{i}(n)+\frac{1}{2}{T}_{j}(n\mathrm{).}$$

By construction, we can obtain the relation easily in the following.

47 $$\begin{array}{ll}{\overline{T}}_{n,total}^{n} & =\,|{\overline{{\rm{\Omega }}}}_{n}|+\sum _{i\in {{\rm{\Omega }}}_{n-1}}[(m+\frac{1}{2}){k}_{i}(n-\mathrm{1)}\times {T}_{i}(n)]\\  & =\,|{\bar{{\rm{\Omega }}}}_{n}|+(m+\frac{1}{2})\sum _{i\in {{\rm{\Omega }}}_{n-1}}[{k}_{i}(n-\mathrm{1)}\frac{\mathrm{2(}mr+\mathrm{1)(}\theta +\mathrm{1)}}{2mr+2-\theta -2mr\theta }{M}_{i}(n-\mathrm{1)}]\mathrm{.}\end{array}$$

### Details about leading scalings of ATT for mixed weight-dependent walks

Since 〈*M*〉_*n*_ is the ATT for the standard weight-dependent walks, through the Eqs (39) and (41), we can obtain the exact dependence relation of ATT on the network size and weight factor *r* for the mixed weight-dependent walks as follows:

When *r* ≠ (*m* − 1)/(2*m*) and 0 < *r* ≤ 1,

48 $$\begin{array}{rcl}{\langle T\rangle }_{n} & \approx  & \frac{(mr+\mathrm{1)(}\theta +\mathrm{1)}}{\mathrm{(4}mr+\mathrm{3)(2}mr+2-\theta -2mr\theta )}\times {({N}_{n}-\mathrm{1)}}^{1+\frac{ln\frac{2mr+2}{m+1}}{ln\mathrm{(2}m+\mathrm{2)}}}\\  &  & +\,\frac{\mathrm{(2}m+\mathrm{1)(4}mr+\mathrm{3)(1}-\theta )}{\mathrm{(2}m+\mathrm{2)(4}mr+4-2\theta -4mr\theta )}\mathrm{.}\end{array}$$

When *r* = (*m* − 1)/(2*m*) > 0,

49 $$\begin{array}{rcl}{\langle T\rangle }_{n} & \approx  & \frac{\theta +1}{4mr+4-2\theta -4mr\theta }\times \frac{m+1}{2m+1}\times ({N}_{n}-\mathrm{1)}\\  &  & +\frac{\mathrm{(2}m+\mathrm{1)(4}mr+\mathrm{3)(1}-\theta )}{\mathrm{(2}m+\mathrm{2)(4}mr+4-2\theta -4mr\theta )}\mathrm{.}\end{array}$$

Then, we can know that Eqs (48) and (49) give an accurate ralation of ATT on the network size and weight factor *r*. So, for very large network,

50 $${\langle T\rangle }_{n} \sim \{\begin{array}{ll}{({N}_{n})}^{1+\frac{ln\frac{2mr+2}{m+1}}{ln\mathrm{(2}m+\mathrm{2)}}}, & {\rm{if}}\,r\ne \frac{m-1}{2m}\,{\rm{and}}\,0 < r\le 1,\\ {N}_{n}, & {\rm{if}}\,r=\frac{m-1}{2m} > 0.\end{array}$$
